# Anterior petrosal (Kawase) approach to petroclival meningioma: 2-dimensional operative video

**DOI:** 10.3171/2022.1.FOCVID21259

**Published:** 2022-04-01

**Authors:** Eva M. Wu, David Altshuler, Stephanie H. Chen, Jacques J. Morcos

**Affiliations:** Department of Neurological Surgery, University of Miami, Miami, Florida

**Keywords:** petroclival meningioma, Kawase, anterior petrosal

## Abstract

Petroclival meningiomas are challenging lesions that can be treated with several surgical approaches. The authors present a 66-year-old woman with a 1.6-cm left petroclival meningioma that was initially observed and then radiated after it grew 8 years later. Despite radiation, the tumor continued to grow to 4 cm; therefore, the patient was referred to the authors’ institution. A left anterior petrosal (Kawase) approach was performed. Postoperatively, the patient had transient cranial nerve IV and VI palsy that improved. The case presentation, surgical anatomy, operative technique, postoperative course, and different surgical approaches are reviewed. The patient gave verbal consent for participating in the surgical video.

The video can be found here: https://stream.cadmore.media/r10.3171/2022.1.FOCVID21259

## Transcript

### 0:26 Clinical Presentation.

We are presenting the case of an anterior petrosal Kawase approach to a large left petroclival meningioma. A 66-year-old female had a 16-mm meningioma 8 years ago. It grew and she was treated with radiosurgery at an outside institution, but worsened after the radiosurgery with dysphagia, dysphonia, hyperesthesia of the face, and gait instability. She was then referred to us.

### 0:46 Neuroimaging Findings.

The MRI shows clearly the large lesion with brainstem compression. There is edema in the left midbrain, particularly, but a nice CSF plane around the tumor. The lesion reaches just caudal to the internal auditory canal, indicating the applicability of the Kawase approach.

### 1:07 Rationale, Risks, Benefits, and Steps of the Procedure.

The rationale for the procedure are the large size of the tumor, and the compression, and the symptoms, and failure of radiosurgery. The risks and benefits are listed on this slide. The alternatives of further radiation or multisession radiosurgery and observation were ruled out. The description of the setup in detail is listed on this slide for a left temporal craniotomy and an anterior petrosectomy. This is the angle of surgical trajectory. We like to put a lumbar drain and position the patient supine with the head turned and a question mark incision.

### 1:54 Identification of Middle Fossa Landmarks.

Here we are, having done already the incision and the craniotomy going subtemporally with CSF egress from the lumbar drain, identifying the middle meningeal artery and V3 of the trigeminal nerve, having peeled the dura from posterior to anterior to avoid injury to the geniculate ganglion of the facial nerve. We are coagulating and dividing the middle meningeal artery to further peel the dura more medially. And here we are peeling the dura from over the surface of V3. The goal is to get all the way to the petrous ridge. Here is the course the greater superficial petrosal nerve. We are using a NIM stimulator to stimulate the geniculate ganglion of the facial nerve and again continue the dissection from posterior to anterior. The GSPN lying on top of the petrous carotid artery is well seen. Further mobilization of the dura of the V3 is done here. Now we are ready to place a self-retaining retractor all the way to the petrous ridge. We are schematizing where the superior semicircular canal and GSPN are, with an angle of 120° with that between them, and here is a shading of Kawase’s rhomboid area.

### 3:27 Anterior Petrosectomy.

We begin the drilling with a diamond drill bit in Kawase’s area. The ink on the bone marks the basal turn of the cochlea, which should not be drilled so not to lose hearing. We did not fully drill the internal auditory canal, as this was not necessary in this case. We of course are using intraoperative navigation as well. We now open the dura over the temporal lobe in a transverse manner. The brain is nicely relaxed.

### 4:00 Dividing Superior Petrosal Sinus and Tentorium.

We continue the dural cut toward the superior petrosal sinus. We then open the dura in the posterior fossa in the presigmoid dura or the dura of the postmeatal petrous surface. We are already seeing the tumor from that angle. We obtain more CSF egress, and I try to identify the most caudal portion of the tumor. It’s important to use the NIM stimulator to identify the facial nerve. We are seeing the surface of the brainstem. Now we are ready to complete the cut of the dura by joining the dural cuts. Here we are coagulating the superior petrosal sinus to continue the cut of the temporal lobe dura into the tentorium and into the presigmoid posterior petrous dura. Here we are engaging the tentorium by cutting it from lateral to medial all the way to the tentorial incisura, having looked for the fourth nerve. Now we can see the fourth nerve clearly having made sure it did not get injured during the final cut of the tentorium. Notice that there is no need for self-retaining retraction in the intradural phase of the surgery. Here is a branch of the superior cerebellar artery. Here is again the fourth nerve coursing on the tumor, and we are dissecting it free from the tumor.

### 5:45 Tumor Resection.

The tumor was not suckable, and it was necessary to use the Sonopet to debulk it carefully, making sure we were not injuring any arteries or nerves. After central debulking, the margin of the tumor is folded in and further exposure of the structures around it, such as the basilar artery, which is seen in this view, can be achieved. It is of course essential to follow the basilar artery and its branches and perforators on the back side of the tumor to avoid injuring them. Here is the superior cerebellar artery grooving the top of the tumor, and we are methodically separating it from the tumor. Another expanded view of the basilar artery after more of the tumor was removed, and this is almost the final piece of tumor coming out. The fifth nerve is seen crossing diagonally the surgical space. Now we have achieved a gross-total resection.

### 7:00 CSF Leak Mitigation.

We place some DuraGen on top of the dural defect, which cannot be reapproximated, and replace the bone flap with plates and screws.

### 7:12 Disease Background and Review of Nomenclature.

Petroclival meningiomas make up about 2% of intracranial neoplasms.^1^ They usually arise from the upper two-thirds of the clivus, and they are located medial to the internal auditory meatus and posterior and the trigeminal nerve is posterior to them.^2^ There are different types of meningiomas that could often be confused, and we have a listing of the nomenclature of all meningiomas of this area, with the origin and what happens to the brainstem and the basilar displacement and what cranial nerves are involved.^1,3–6^

### 7:50 Review of Different Approaches to Petroclival Meningiomas.

The surgical approaches that could be considered are also listed with their indications, advantages, and disadvantages—from orbital zygomatic to retrosigmoid to staged retrosigmoid and transylvian.^7–9^ We can then consider the posterior petrosal family of approaches from retrolabyrinthine to partial translabyrinthine to complete translabyrinthine to transotic and, finally, the transcochlear.^6,9^ The posterior petrosal approach is selected when the lesion goes below the internal auditory canal into what we like to designate the third quarter of the clivus.^6,9^ It was not necessary in this particular case. Anterior petrosal approach was what we selected here versus the combined posterior petrosal approach or a complete petrosectomy.^6,9^ The far lateral approach is considered when it is the inferior portion of the clivus. Additionally, one has to remember that the endonasal endoscopic approach is best considered for extradural lesions and not for intradural lesions such as this meningioma.^10^

### 9:16 Review of Clinical and Imaging Outcome.

The histology was a WHO grade I meningioma with a Ki-67 of 2%. The patient had a postoperative cranial nerve IV and VI palsy. The cranial IV palsy recovered very quickly and the cranial nerve VI palsy was still in the process of recovering at the time of this report. Postoperative MRI showed complete resection of the tumor without any stroke or other issues. Thank you.

## Disclosures

The authors report no conflict of interest concerning the materials or methods used in this study or the findings specified in this publication.

## Author Contributions

Primary surgeon: Morcos. Assistant surgeon: Altshuler, Chen. Editing and drafting the video and abstract: Morcos, Wu, Altshuler. Critically revising the work: all authors. Reviewed submitted version of the work: all authors. Approved the final version of the work on behalf of all authors: Morcos.
